# Impact of Ferrite–Cementite Microstructure on Tensile and Cutting Behaviour of C75S Steel

**DOI:** 10.3390/ma19091836

**Published:** 2026-04-29

**Authors:** Jarosław Kaczmarczyk

**Affiliations:** Department of Theoretical and Applied Mechanics, Faculty of Mechanical Engineering, Silesian University of Technology, 18A Konarskiego Street, 44-100 Gliwice, Poland; jaroslaw.kaczmarczyk@polsl.pl; Tel.: +48-322372790

**Keywords:** micro-scale finite element method, ferrite–cementite interface, tensile test, cutting process, SEM analysis

## Abstract

In this study, scanning electron microscopy (SEM) analysis is used to reveal the real microstructure of C75S steel and to compare grain morphology and deformation features with numerical predictions. A micro-scale finite element model of C75S steel is developed to investigate its tensile response in order to understand how steel actually deforms and fails at the microstructure level. Subsequently, the validated microstructural model is employed to simulate the cutting process using the finite element method, focusing on stress concentration and damage initiation at the grain and interface zones. The results demonstrate that microstructural modelling provides improved insight into deformation and fracture mechanisms compared to homogenised approaches, highlighting the critical role of cementite distribution and interfacial behaviour during tensile loading and micro-scale cutting. The cementite particle sizes in C75S steel range from approximately 0.5 to 2.0 µm, with circularity values between 0.7 and 0.95 and a volume fraction of about 10–12%. The proposed framework offers a robust basis for predicting the cutting performance of high-carbon steels.

## 1. Introduction

High-carbon steels such as C75S are widely used in a variety of applications including cutting tools [1,2], springs [3,4], and precision mechanical components requiring high strength [5], wear resistance [6], and dimensional stability [7,8]. The mechanical and machining behaviour of these steels is strongly governed by their microstructural characteristics, particularly the morphology, distribution and interaction of ferrite and cementite phases [9,10,11]. As manufacturing processes increasingly move toward miniaturisation and high-precision machining, understanding material behaviour at the micro-scale is essential [12,13].

In recent years, significant effort has been devoted to numerical–experimental analysis of guillotine cutting of cold-rolled steels and sheet bundles. Shao et al. numerically investigated the mechanical response of defective tunnel linings and demonstrated how detailed material modelling and strengthening layers influence local stress and damage fields in concrete–steel systems [14]. For metallic sheets, Kaczmarczyk and co-workers developed finite element models of ultra-thin sheet bundles and analysed the cutting process with defined tool geometries, showing that the fracture surface consistently divides into ductile and brittle zones separated at about 1/5–1/3 of the sheet thickness and that front/back knife sides exhibit different plastic zones and edge defects [15,16,17]. In C75S cold-rolled steel, the cutting simulations used a bilinear elastic–plastic model calibrated for a spheroidised microstructure consisting of a ferritic matrix with globular cementite particles, while experimental SEM observations confirmed the distinct brittle and ductile regions along the cut edge [17]. Follow-up work on single-sheet cutting further validated these dynamic FEM models and highlighted the importance of failure criteria and mesh design for accurately predicting crack initiation and propagation during guillotine cutting [18]. Together, these contributions establish a robust macroscopic description of C75S sheet separation, but they treat the steel as an effectively homogeneous continuum and do not explicitly resolve individual cementite particles, ferrite grains or ferrite–cementite interfaces under micro-scale loading, damage initiation, and interface-driven fracture [19,20].

In high-carbon steels, the strong mechanical contrast between the ductile ferritic matrix and the brittle cementite phase plays a decisive role in deformation and failure mechanisms, especially under high strain and strain-rate conditions typical for cutting processes [21]. In this study, Bergs et al. present the in-process analysis of the strain rate and strain in the primary shear zone using high speed Digital Image Correlation (DIC) techniques at the macroscale. The comparison of measured and computed results shows the suitability of the DIC techniques and the robustness of the modelling approaches.

Foundational concepts of the finite element method (FEM), contact formulations, and their implementation in commercial codes provide the numerical basis for simulating complex deformation and tool–workpiece interaction [22,23,24]. Building on these notions, standard references on linear and nonlinear finite element analysis for continua and solid mechanics [24,25,26] supply the variational framework, element technology, and solution strategies used here for micro-tensile and micro-cutting simulations. Latest advances in finite element analysis and microstructural characterisation techniques [27] have enabled researchers the modelling of individual grains and phases [28,29,30]. Micro-scale FEM approaches, where ferrite and cementite are represented as distinct material domains, offer a powerful tool to investigate phase-specific deformation, stress concentration, and damage evolution [31,32,33]. In this context, the ferrite–cementite interface is of particular importance, as it governs load transfer between phases and often acts as a preferred region for crack initiation and propagation [19,20].

Experimental techniques such as scanning electron microscopy (SEM) provide detailed insight into the actual grain morphology and phase distribution in steels like C75S. Integrating SEM-based observations into numerical models allows researchers to capture more realistic representation of the microstructure and enables them direct qualitative and quantitative validation of simulation results. Such combined experimental–numerical approaches are especially valuable for studying micro-cutting, where tool–material interaction occurs at length scales comparable to the grain size [34,35].

Before addressing the complexity of cutting simulations, it is essential to validate the micro-scale material model under simpler loading conditions. Tensile testing represents a fundamental benchmark for assessing the accuracy of constitutive laws, phase interaction models, and interface behaviour. A successful reproduction of tensile response at the micro-scale provides confidence in the predictive capability of the model when applied to more complex processes such as cutting [36,37].

Therefore, the objective of this study is to develop a micro-scale finite element model of C75S steel that explicitly takes into account ferrite matrix, cementite phase, and interface between ferrite and cementite. The modelling strategy is first validated through microstructural tensile simulations, focusing on stress–strain behaviour and phase-level deformation mechanisms. Subsequently, the validated material model is employed to simulate the cutting process using the finite element method, with particular emphasis on stress concentration, and damage evolution.

This work aims at contribution to a deeper understanding of the role of microstructure in the tensile tests of high-carbon steels and provides a robust numerical framework for predicting micro-scale cutting phenomena in C75S steel. In contrast to conventional approaches that rely on homogenised material descriptions or simplified particle geometries without explicit phase boundaries, this work introduces a microstructure-resolved model of spheroidised C75S steel with well-defined ferrite–cementite interfaces, enabling a coupled investigation of tensile and micro-cutting behaviour and provides new insight into phase interaction mechanisms.

## 2. Materials and Methods

This chapter describes the material characterisation and numerical methods applied in the modelling process. The investigated material was C75S steel (Interbelts S.C., Wrocław, Poland), whose microstructure and chemical composition were examined using scanning electron microscopy (SEM) with a cold field emission (FESEM) HITACHI S-4700 (Hitachi, Ltd., Tokyo, Kanto, Japan) equipped with the energy-dispersive X-ray (EDS) NORAN Vantage spectrometer (Noran C). In addition, numerical simulations based on the finite element method (FEM) were performed to investigate the mechanical behaviour of the material at the micro-scale, with particular emphasis on microstructural modelling using an implicit solution scheme [38,39].

### 2.1. Material Properties of High-Carbon Steel C75S

The investigated material was high-carbon steel C75S, which is commonly used in applications requiring high strength and wear resistance [40]. The steel is characterised by a carbon content of approximately 0.70–0.80 wt.% together with a small addition of other elements that have been presented in detail (Table 1) and is based on the EN 10132-4 standard [41].

In the present work, the microstructure was represented using a simplified multiphase approach. The material was modelled as a composite consisting of three constituents:

- ferritic matrix,
- globular cementite particles,
- an interfacial transition zone between ferrite and cementite.

Ferrite represents the ductile phase responsible for the plastic deformation of the material, while cementite acts as a hard and brittle reinforcing phase. The interfacial region was introduced in order to account for stress and strain localisation phenomena that typically occur at phase boundaries and may influence crack initiation or damage evolution during deformation. The mechanical properties of individual phases are juxtaposed in Table 2.

The modelling technique was applied for materials with a composite microstructure, in which the phases have been modelled with different hardening behaviour. The ferritic phase constituted the soft matrix, in which the much harder globular cementite grains are dispersed. It was assumed that the cementite grains are surrounded by an interfacial thin layer with mechanical properties intermediate between ferrite and cementite. All these phases have been modelled as deformable elastically as well as plastically until reaching the limited strain value corresponding to the uniform elongation of the sample, which has been established during experimental investigation on the level of strain equal to 0.15. The material properties of ferrite, cementite and the intermediate phase were estimated on the basis of available literature [27,28,29,42]. The relationship between the true stress–strain curve of the ferritic matrix and the interface with different mechanical stability has been assumed according to the following mathematical formula [42]:

(1) $$\sigma =R_{e}\cdot {\left(1+H\cdot \varepsilon \right)}^{n},$$

where *R_e_*—yield strength, *H*—hardening modulus, *ε*—true strain, and *n*—strain hardening exponent.

**Figure 1:**
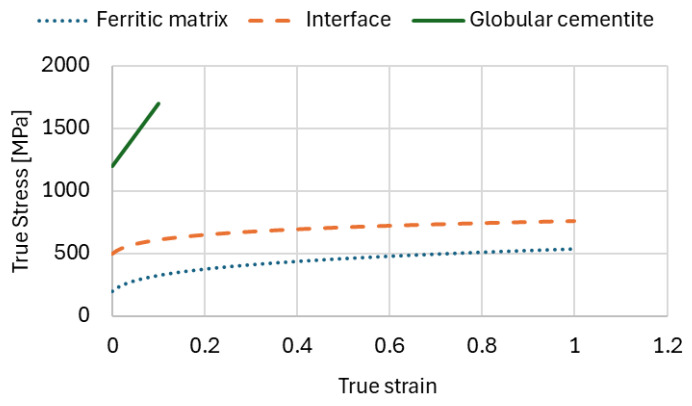
Mechanical properties of individual structural constituents of C75S steel.

All aforementioned material phases described by Equation (1) are additionally shown in Figure 1.

Different constitutive models were assigned to each phase to reflect their distinct mechanical properties. The ferrite phase was modelled as an elastic–plastic material capable of undergoing significant plastic deformation, whereas cementite was assumed to behave as a relatively stiff and brittle phase which undergoes minor plastic deformation. It was modelled as a high-strength, nearly linear phase to reflect its brittle nature characterised by a tangent modulus equal to 5 GPa. This representation captures the high stiffness and low ductility of cementite, which strongly influences stress concentration and crack initiation.

The interfacial zone was assigned intermediate mechanical properties to capture possible strain and stress localisation effects at the phase boundaries.

### 2.2. Microstructural Model for Numerical Tensile Test

**Figure 2:**
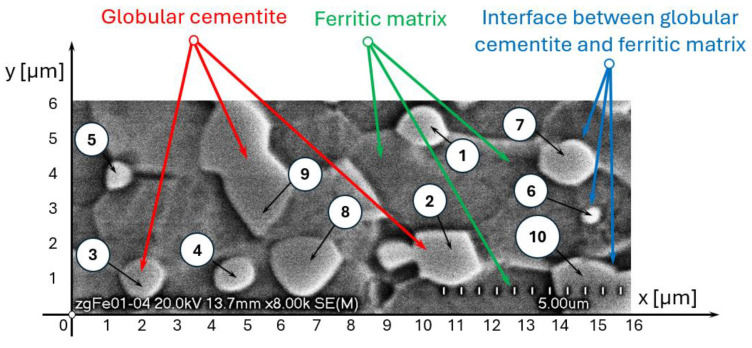
The initial microstructure of investigated C75S steel with numbered grains of globular cementite from 1 to 10.

The microstructure of C75S steel consists of a ferritic matrix containing globular cementite particles separated by a thin interfacial transition zone. The microstructural observations were performed using a scanning electron microscope with a cold field emission (FESEM) HITACHI S-4700 (Hitachi, Ltd., Tokyo, Japan) equipped with the energy-dispersive X-ray (EDS) NORAN Vantage spectrometer (Noran Co., Vernon, CA, USA) at a magnification of 8000×. The microstructure of C75S steel presented in Figure 2 reveals ten spheroidised cementite particles distributed within a ferritic matrix. The cementite particles exhibit a globular morphology with grain sizes of approximately 0.5–2 µm. The matrix phase is primarily ferritic, and well-defined ferrite–cementite phase boundaries can be observed.

**Figure 3:**
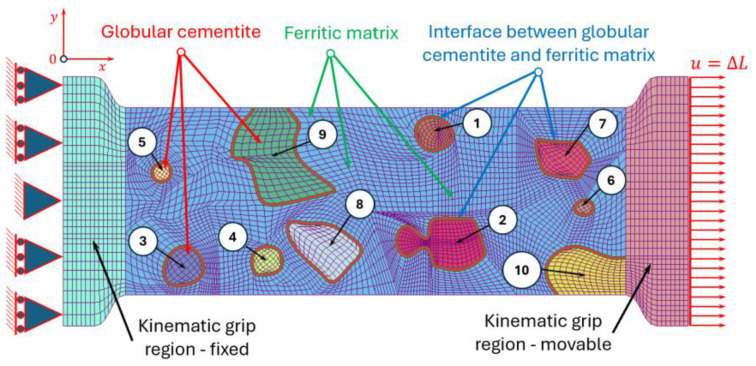
Model of meshed sample representing microstructural constituents of C75S steel with numbered grains of globular cementite from 1 to 10 for uniaxial tensile test.

Based on the observed microstructure, a corresponding numerical model was established using the finite element method in the LS-DYNA system developed by Livermore Software Technology Corporation (LSTC, Livermore, CA, USA) and part of Ansys after the 2019 acquisition. The analysed domain was assumed as a deformable body under a plane strain state and discretised using two-dimensional finite elements. This approach enabled an accurate representation of the phase distribution and the mechanical interaction between ferrite, cementite, and the interfacial region (Figure 3). The model should be interpreted as a representative microstructural configuration aimed at capturing deformation and fracture mechanisms rather than the exact phase proportions.

The computational domain was discretised using quadrilateral and triangular finite elements with four and three nodes, respectively. Each node possessed two translational degrees of freedom corresponding to displacements along the *x*- and *y*-axes. The mesh density was selected in a such a way that would ensure adequate representation of phase boundaries and to capture stress and strain gradients occurring near the interfaces.

**Figure 4:**
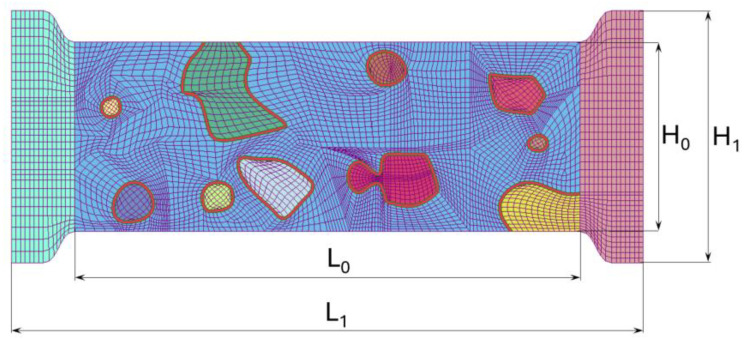
Model of meshed sample representing microstructural specimen dimensions of C75S steel for uniaxial tensile test.

The geometrical model used for the micro-scale uniaxial tensile test is presented in Figure 4, while the corresponding dimensions are summarised in Table 3.

The analysed domain represents a microstructural specimen composed of a ferritic matrix containing ten globular cementite grains surrounded by an interfacial transition zone.

In order to reproduce the boundary conditions of the tensile test, the deformable microstructural region was extended by two kinematic grip regions (Figure 3). These grips were modelled as perfectly rigid bodies and attached to both ends of the specimen by means of common nodes between the deformable zone and the rigid one. One grip was fully constrained, while the opposite grip was allowed to move along the loading direction (*x*-axis), enabling the application of controlled displacement. This configuration ensures a uniform transfer of deformation to the microstructural domain during the simulation.

The discretisation parameters of the numerical model representing the micro uniaxial tensile test, including the number of elements and nodes assigned to individual components, are juxtaposed in Table 4.

The deformable sheet representing the microstructure of C75S steel was discretised using predominantly four-node quadrilateral elements, with a limited number of triangular elements introduced to capture geometrical complexity at phase boundaries. The rigid grips were modelled using a coarser mesh, as their deformation was neglected. The total number of elements and nodes reflects the combined discretisation of both deformable and rigid parts of the model.

The microstructural geometry was generated in such a way that cementite particles were distributed within the ferritic matrix while maintaining the prescribed phase fractions (Table 5). The areas and centroids of the cementite grains were calculated to describe their spatial distribution within the ferritic matrix and to assess their influence on strain and stress localisation.

The obtained values, together with the corresponding areas of the ferrite matrix and interfacial zones, are summarised in Table 6. Furthermore, the percentage contribution of the individual constituents, providing a quantitative representation of the phase distribution within the analysed microstructural domain is also presented.

The fraction and spatial distribution of the phases were defined based on metallographic observations of the steel microstructure (Figure 2).

The developed microstructural model serves as a preliminary stage for further simulations of material separation processes at the micro-scale. In particular, the tensile simulations were used to verify the ability of the model to reproduce realistic deformation mechanisms prior to implementing more complex loading conditions associated with the cutting process.

The objective of the present study is not to reproduce the exact statistical representation of the real microstructure, but rather to investigate the local mechanical response and interaction mechanisms at the micro-scale. In this context, the selected number of particles is sufficient to capture the key phenomena, such as stress localisation, load transfer, and interface effects, while avoiding excessive computational cost.

Furthermore, the use of a two-dimensional model is a commonly adopted approach in micro-mechanical simulations, particularly in preliminary or mechanism-focused studies. While it does not fully capture three-dimensional effects, it provides some quantitative insights into the deformation behaviour as well as equivalent Huber–Mises strain and stress values.

Nevertheless, the limitations associated with these assumptions are acknowledged. In particular, the reduced number of particles may not fully represent the statistical variability of the microstructure, and the 2D formulation neglects out-of-plane interactions.

### 2.3. Microstructural Model for Cutting Simulation

**Figure 5:**
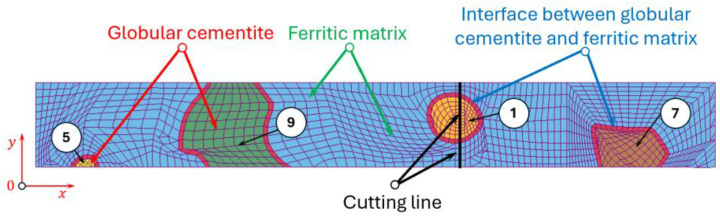
Model of meshed sheet representing microstructural constituents of C75S steel with selected numbered grains of globular cementite for micro-scale cutting process.

The micro-scale cutting process of C75S steel was investigated using a two-dimensional numerical model developed within the framework of the finite element method. The analysed microstructural domain consisted of deformable sheet with dimensions of 16 μm in length and 2 μm in height, containing several globular cementite particles embedded in a ferritic matrix and separated by an interfacial transition zone. One cementite particle was intentionally located along the predefined cutting line (Figure 5).

**Figure 6:**
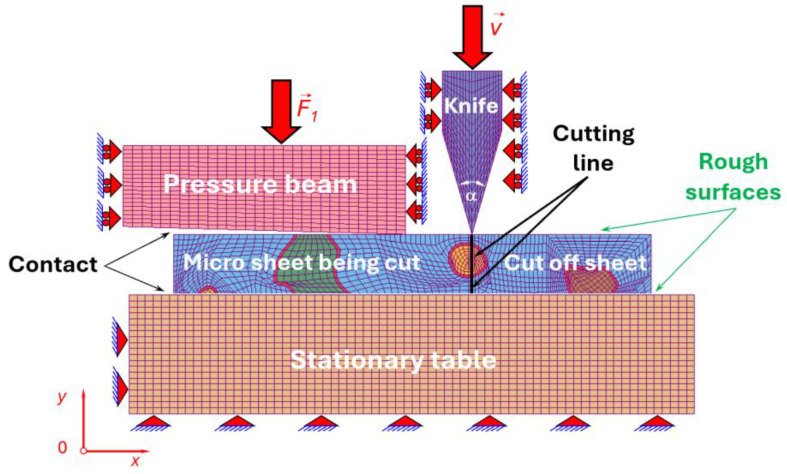
Model of meshed sheet representing microstructural constituents of C75S steel with selected grains of globular cementite for micro-scale cutting process.

The sheet was placed on a stationary rigid table and constrained by a pressure beam applying a relatively low normal force to prevent excessive out-of-plane motion. A rigid cutting tool moved vertically downwards with a low velocity in order to capture the quasi-static material response during the cutting process. The tool penetrated the material until reaching the upper surface of the stationary table and subsequently returned to its initial position (Figure 6).

In the present study, the friction coefficient was based on values commonly reported in the literature for steel–tool interactions under dry or simplified contact conditions. The aim was to adopt a representative value rather than to model a specific cutting configuration. Contact interactions between the deformable sheet and the perfectly rigid bodies (stationary table, pressure beam, and cutting tool) were defined using Coulomb–Moren dry friction with a static coefficient of 0.2 and a kinetic coefficient of 0.1 [43].

The model of micro sheet was formulated under plane state of strain and discretised using mainly four nodal quadrilateral elements together with a relatively small number of triangular elements possessing two translational degrees of freedom per node.

The discretisation parameters of the numerical model representing the micro-cutting process, including the number of elements and nodes assigned to individual components, are presented in Table 7.

The deformable microstructural domain was discretised with a fine mesh to accurately capture stress and strain gradients within individual phases, particularly in the vicinity of interfaces and along the cutting line. The mesh composed of predominantly four-node quadrilateral elements was used, while a limited number of triangular elements were introduced to accommodate geometric complexity. The rigid components, including the stationary table, pressure beam, and cutting tool were modelled using slightly coarser mesh, as their deformation was neglected.

The current modelling scope is based on a quasi-static, isothermal assumption, which was intentionally adopted to isolate and analyse the fundamental microstructural mechanisms, such as stress distribution, load transfer, and phase interaction between ferrite and cementite. This limitation and the corresponding range of applicability show that the model should be interpreted as a tool for identifying fundamental deformation mechanisms rather than a statistically representative description of the microstructure.

The evolution of the cutting process was analysed applying the distributions of equivalent Huber–Mises strain and stress presented for several successive stages of deformation in the next chapter.

## 3. Results and Discussion

In this chapter, the experimental and numerical results are presented and discussed. The microstructural characterisation of C75S steel was performed using scanning electron microscopy (SEM) combined with energy-dispersive X-ray spectroscopy (EDS). In addition, the results of numerical simulations based on the finite element method (FEM) are analysed, including the micro-scale uniaxial tensile test and the micro-scale cutting process, in order to evaluate the evolution of stress and strain zones within the material microstructure.

### 3.1. SEM–EDS Analysis of the Microstructure

The microstructure of the investigated C75S steel was examined using scanning electron microscopy coupled with energy-dispersive X-ray spectroscopy in order to determine the elemental composition of individual microstructural constituents. All samples were analysed at the same:

- take-off angle equal to 31.7 deg.,
- time of 50 s,
- acceleration voltage 20 keV.

**Figure 7:**
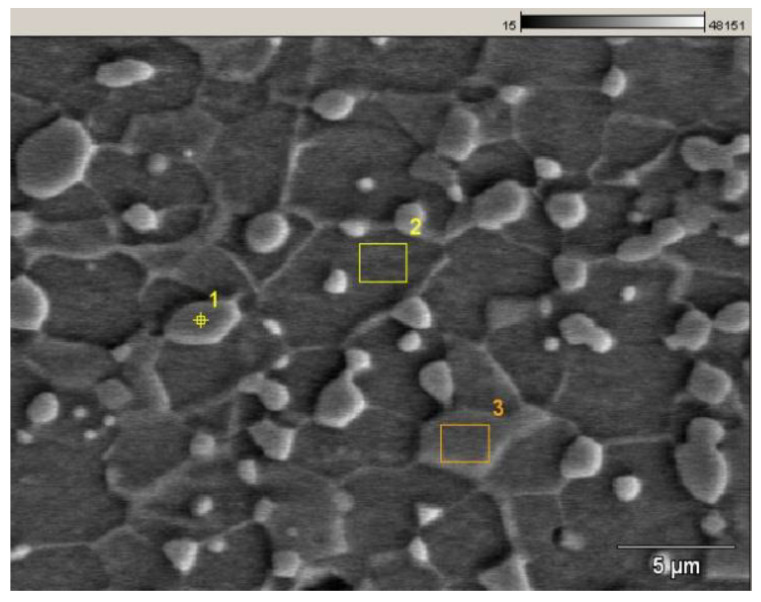
Three characteristic areas of the microstructure: (1) a point located inside a globular cementite particle, (2) a small rectangular region representing the ferritic matrix, (3) a small rectangular region corresponding to globular cementite.

The analyses were performed on selected regions corresponding to ferrite and globular cementite particles (Figure 7). The measurement locations were selected to ensure phase-specific compositional analysis while minimising interaction volume effects. The point analysis was placed at the centre of a cementite particle to avoid signal contribution from the ferritic matrix. The rectangular areas were used to obtain averaged compositions of both ferrite and cementite, improving statistical reliability and reducing local variability. All regions were carefully positioned away from phase boundaries to ensure phase purity of the measurements.

The obtained spectra were interpreted on the basis of the detected characteristic X-ray lines.

#### 3.1.1. Point Analysis of Globular Cementite

The EDS point analysis performed at the location corresponding to a globular particle revealed the presence of the following characteristic X-ray lines:

- Fe–K,
- Mn–K,
- Cr–K.

The dominant signal originates from Fe–K radiation, indicating that iron is the principal component of the analysed phase. This observation is consistent with the presence of cementite (Fe_3_C) particles embedded in the ferritic matrix.

**Figure 8:**
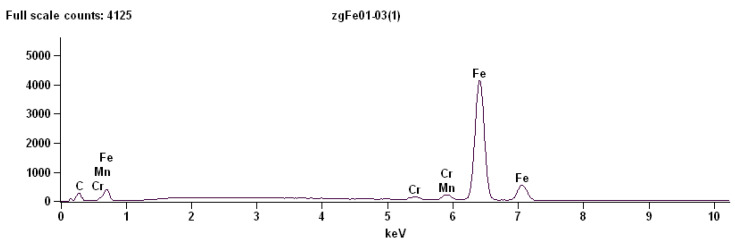
The energy-dispersive X-ray spectroscopy (EDS) for a point located inside a globular cementite particle.

The detection of Mn–K and Cr–K signals indicates the presence of manganese and chromium in the analysed particle (Figure 8). These elements occur commonly in small quantities in carbon steels and may partially substitute iron atoms in the cementite lattice. Such substitution is well documented in alloyed or impurity-modified cementite, where alloying elements segregate preferentially to carbide phases.

Table 8 provides the quantitative results of the energy-dispersive X-ray spectroscopy analysis performed at a point located at a globular cementite particle in C75S steel.

The enrichment of manganese and chromium in cementite compared to ferrite is frequently observed due to the higher solubility of these elements in carbides. Their presence may influence the stability and mechanical properties of the cementite particles, particularly their hardness and resistance to deformation.

#### 3.1.2. Area Analysis of the Ferritic Matrix

**Figure 9:**
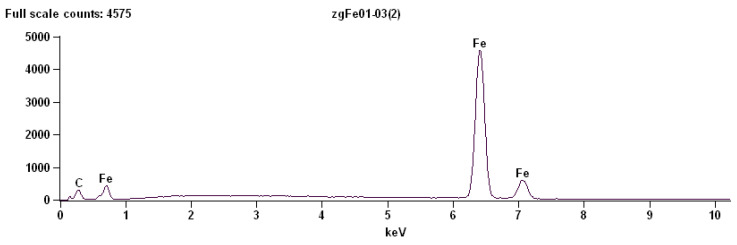
The energy-dispersive X-ray spectroscopy (EDS) for a small rectangular region representing the ferritic matrix.

The EDS analysis performed on a small rectangular region representing the ferritic matrix showed primarily the Fe–K characteristic peak (Figure 9).

Ferrite is the a-iron phase, which consists almost entirely of iron with very limited solubility for carbon and other alloying elements. Therefore, the predominance of the Fe–K peak confirms that the analysed region corresponds to the ferritic phase forming the matrix of the microstructure.

Table 9 provides the quantitative results of the energy-dispersive X-ray spectroscopy analysis performed for a small rectangular region representing the ferritic matrix in C75S steel.

The absence or very low intensity of additional elemental peaks suggests that alloying elements are either present in very small concentrations or preferentially segregated to other microstructural constituents such as carbides.

#### 3.1.3. Area Analysis of Globular Cementite

The EDS analysis of the rectangular region corresponding to a globular cementite particle revealed the presence of the following characteristic peaks:

- Fe–K,
- Mn–K,
- W–L.

**Figure 10:**
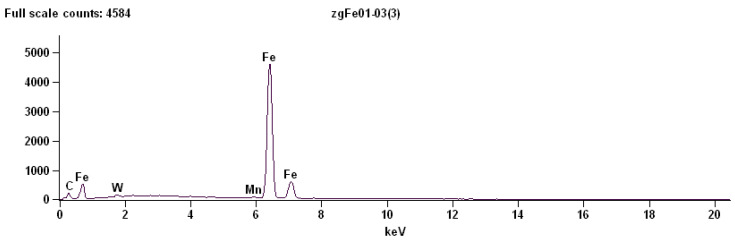
The energy-dispersive X-ray spectroscopy (EDS) for a small rectangular region corresponding to globular cementite.

The Fe–K peak again confirms that the analysed particle is primarily composed of iron. The presence of Mn–K radiation indicates manganese enrichment within the carbide phase, which is consistent with the tendency of manganese to partition into cementite (Figure 10).

Table 10 provides the quantitative results of the energy-dispersive X-ray spectroscopy analysis performed for a small rectangular region corresponding to globular cementite in C75S steel.

The enrichment of manganese in cementite compared to ferrite is frequently observed due to the higher solubility of this element in carbides. An additional signal corresponding to the W–L line was detected in this region. Tungsten is not a typical alloying element in C75S steel, which suggests that its presence may originate from external sources. One possible explanation is contamination introduced during specimen preparation, for example from tungsten-containing polishing media or from wear of tungsten-based tools used during cutting or grinding of the sample.

#### 3.1.4. Discussion of SEM-EDS Analysis of the C75S Steel Microstructure

The SEM–EDS results confirm the heterogeneous microstructure of the investigated C75S steel consisting of a ferritic matrix with dispersed globular carbide particles. The ferritic phase is characterised by a dominant iron signal, whereas the carbide particles show additional enrichment in alloying elements such as manganese and chromium.

The preferential segregation of alloying elements into cementite may influence the mechanical behaviour of the material by modifying the hardness and brittleness of the carbide phase as well as the strength of the ferrite–cementite interface. These microstructural characteristics are particularly important for numerical simulations of the material performance at the micro-scale, where the presence of chemically heterogeneous phases may contribute to stress and strain localisation.

### 3.2. Micro-Scale Tensile Test Simulation

The objective of this subchapter was to evaluate the evolution of equivalent Huber–Mises stress and strain distributions and to identify potential locations of stress concentration and crack initiation within the microstructure of C75S steel using the finite element method in the LS-DYNA system [22]. The analysed sample was modelled as a plane state of strain and treated as a deformable body connected to kinematic grip regions at both ends. These grip regions were assumed to be perfectly rigid, with one end fully constrained while the opposite end was allowed to move along the *x*-axis in order to apply the tensile displacement.

#### 3.2.1. Strain Distribution

The numerical calculations were carried out using LS-DYNA with an implicit solution scheme [22]. The total duration of the analysis was defined as one pseudo second, which represents a numerical loading parameter rather than real physical time.

**Figure 11:**
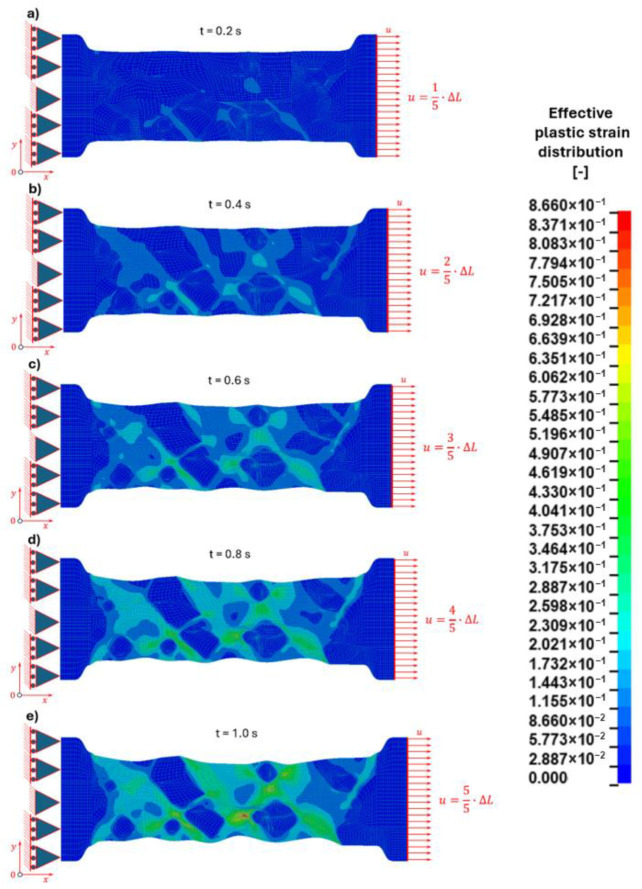
Effective plastic strain distribution in the microstructural constituents during the progressive uniaxial tensile test for the chosen pseudo time instances [s]: (**a**) t = 0.2, (**b**) t = 0.4, (**c**) t = 0.6, (**d**) t = 0.8, (**e**) t = 1.0.

The distribution of equivalent strains reveals a heterogeneous deformation pattern within the microstructure due to the strong mechanical contrast between the ferritic matrix and the cementite particles (Figure 11).

The applied displacement corresponded to the total plastic displacement obtained from experimental tensile testing performed at the macro-scale. This value was determined by proportional scaling of the experimentally measured displacement at failure and subsequently applied to the microstructural model. In order to analyse the progressive development of deformation, the total displacement equal to 2.5 μm was divided into five loading stages corresponding to:

- 1/5 of the total displacement (Figure 11a),
- 2/5 of the total displacement (Figure 11b),
- 3/5 of the total displacement (Figure 11c),
- 4/5 of the total displacement (Figure 11d),
- 5/5 of the total displacement—final stage (Figure 11e).

For each stage, the distribution of equivalent strain according to the Huber–Mises hypothesis was evaluated.

At the initial loading stage (1/5 of the total displacement—Figure 11a), plastic deformation is mainly localised in the ferritic matrix. The cementite particles remain largely undeformed due to their significantly higher stiffness and strength compared to the surrounding ferrite. Elevated strain concentrations appear at the interfaces between the ferritic matrix and the cementite grains, indicating the onset of localised deformation.

As the applied displacement increases (2/5 and 3/5 of the total displacement—Figure 11b,c), the strain localisation becomes more pronounced. The ferritic matrix accommodates most of the plastic deformation, while the interfaces surrounding the cementite particles act as preferred locations for strain accumulation. These regions represent potential sites for the initiation of microcracks due to the differences in deformation between the ductile ferrite and the brittle carbide phase.

At higher loading stages (4/5 of the total displacement—Figure 11d), the strain concentrations intensify and extend along the ferrite–cementite interfaces. In some cases, localised areas of high equivalent strain occur within the cementite particles themselves, particularly for larger grains.

At the final stage (5/5 of the total displacement—Figure 11e), the maximum strain values are observed in the ferritic matrix adjacent to the cementite particles and within selected cementite grains. The results indicate that the location of strain localisation strongly depends on the size of the cementite particles.

For smaller cementite grains, the highest strain values appear primarily at the ferrite–cementite interface, indicating that microcrack initiation is likely to occur at the interfacial region. In contrast, for larger cementite particles, significant strain localisation develops within the interior of the cementite grain, which may lead to crack initiation inside the carbide phase.

#### 3.2.2. Stress Distribution

**Figure 12:**
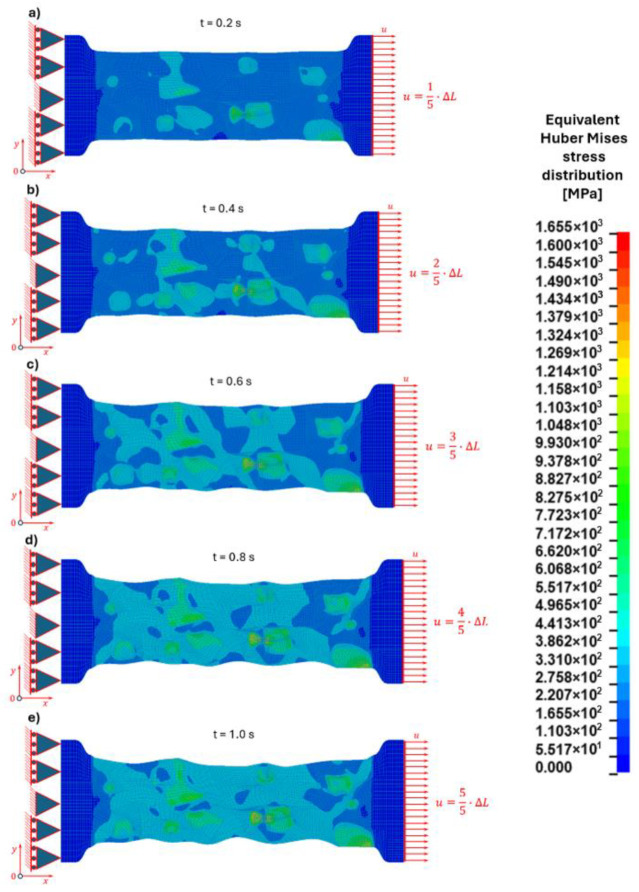
Equivalent Huber–Mises stress distribution in the microstructural constituents during the progressive uniaxial tensile test for the chosen pseudo time instances [s]: (**a**) t = 0.2, (**b**) t = 0.4, (**c**) t = 0.6, (**d**) t = 0.8, (**e**) t = 1.0.

The distribution of equivalent stresses follows a similar heterogeneous pattern reflecting the differences in mechanical properties between the phases (Figure 12).

At the early loading stage (1/5 of the total displacement—Figure 12a), stress concentrations appear near the cementite particles due to the constraint imposed by the stiff carbide phase on the surrounding ferritic matrix. The ferrite matrix experiences lower stress levels compared to the cementite particles.

With increasing deformation (2/5 and 3/5 of the total displacement—Figure 12b,c), the stress distribution becomes increasingly non-uniform. The cementite particles carry a significant portion of the applied load due to their higher stiffness, resulting in elevated stress levels within these particles. Simultaneously, stress concentrations develop at the ferrite–cementite interfaces.

At advanced loading stages (4/5 and 5/5 of the total displacement—Figure 12d,e), the highest stress values are observed either within the cementite particles or at their interfaces with the ferritic matrix. The precise location of the maximum stress depends on the size of the particle.

For smaller cementite particles, the maximum stress occurs primarily along the ferrite–cementite interface, which indicates that the interface acts as a critical region for damage initiation. Conversely, for larger cementite grains, the highest stresses are concentrated in the central region of the particle, which may cause crack initiation within the brittle cementite phase.

#### 3.2.3. Discussion of Micro-Scale Tensile Test Simulation

The numerical results demonstrate that the mechanical response of the microstructure is strongly influenced by the size of the cementite particles and the mechanical interaction between the phases. The mismatch in deformation behaviour between the ductile ferritic matrix and the brittle cementite phase leads to significant stress and strain localisation (Table 11).

During the progressive loading, the equivalent Huber–Mises stresses increase in all phases. The highest stress levels are consistently observed in the cementite particles, reaching 1655 MPa, followed by the interface (703 MPa) and the ferritic matrix (478 MPa). This distribution is governed by the load transfer mechanism, where the softer ferritic matrix undergoes plastic deformation and transfers load to the stiffer and stronger cementite phase, leading to stress accumulation within the particles. The interface acts as a transition region, redistributing stresses between the phases and accommodating mechanical incompatibilities.

The strain distribution exhibits an inverse trend. The ferritic matrix experiences the highest equivalent strain (0.866), indicating its dominant role in plastic deformation. The interface shows intermediate strain values (0.661), while the cementite particles undergo only limited deformation (0.084), reflecting their high stiffness and predominantly elastic response. This contrast confirms that the overall deformation is governed by plastic flow in the ferritic matrix, while cementite primarily acts as a load-bearing phase.

A more detailed analysis of stress distribution within individual cementite particles reveals a strong dependence on particle size. For smaller globular cementite grains (0.5–1.2 µm), the highest equivalent Huber–Mises stresses are localised near the particle boundaries, indicating that the interface region is the most critical site for stress concentration. For example, in grain number 1 (Figure 2 and Figure 3), the maximum stress at the centre reaches 524 MPa, whereas at the boundary it increases to 803 MPa. This suggests that damage initiation is likely to occur at or near the interface.

In contrast, for larger cementite grains (approximately 1.2–2 µm), the stress distribution shifts, with the highest stresses occurring in the particle core and lower values at the boundaries. For instance, in grain number 2 (Figure 2 and Figure 3), the maximum stress at the centre is 612 MPa, compared to 583 MPa at the boundary. This behaviour leads to a more uniform stress distribution and a tendency for internal stress accumulation within the particle.

These results indicate that the mechanism of damage initiation in cementite is size-dependent. Smaller particles are more prone to interface-controlled failure, while larger particles may exhibit a higher likelihood of internal cracking. Overall, the interplay between particle size, load transfer, and deformation incompatibility governs the local stress state and potential failure mechanisms during tensile loading.

#### 3.2.4. Comparison of Numerical and Experimental Results

**Figure 13:**
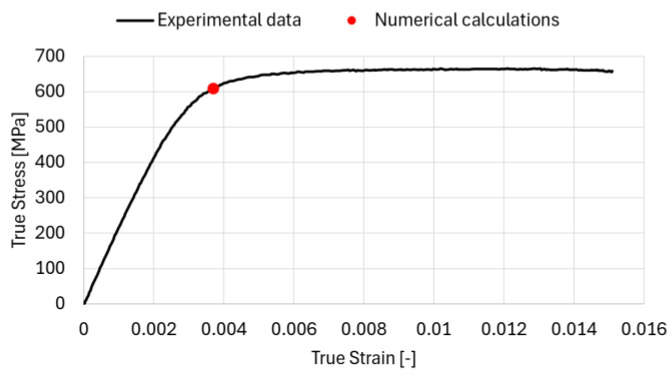
Comparison of the true stress versus true strain obtained from experimental data and yield strength from numerical calculation.

The experimental investigations to support the numerical analysis were carried out, with particular focus on the true stress–true strain behaviour. Tensile tests were performed using a Zwick testing machine. A quantitative comparison of numerical results with experimental data has been elaborated. In particular, the experimental true stress versus true strain response as well as yield strength obtained from numerical calculation have been directly compared (Figure 13).

Microstructure of the steel is composed of ferrite and cementite phases, and its mechanical properties are the result of the interaction between these two phases. The strength of ferrite–cementite steels can be considered to follow the proposition similar to the one elaborated by Gladman et al. [44,45].

(2) $$\sigma_{y}=A_{f}^{m}\cdot \sigma_{f}+\left(1-A_{f}^{m}\right)\cdot \sigma_{c},\;$$

where $\sigma_{y}$ is yield strength of ferrite–cementite aggregate, $\sigma_{f}$ is yield strength of ferrite, $\sigma_{c}$ is yield strength of cementite, $A_{f}$ is area fraction of ferrite, and *m* is a constant (1.95) which incorporates the nonlinear dependence of $\sigma_{y}$ on area fractions of the constituents.

**Figure 14:**
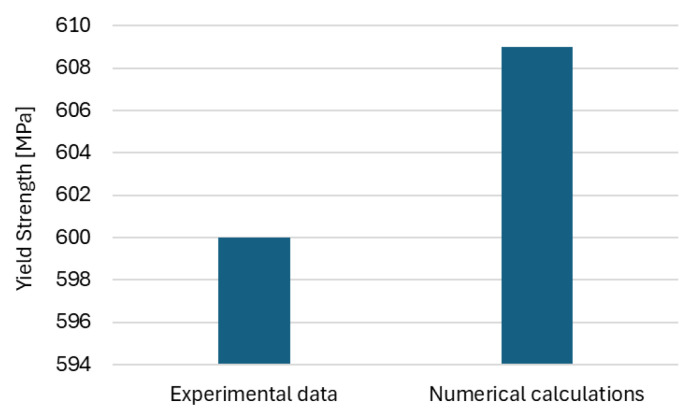
Comparison of the yield strength obtained from experimental measurement and numerical calculation.

The evaluated yield strength of the modelled microstructure on the basis of Equation (2) is equal to ca. 609 MPa which corresponds to the yield strength of the investigated C75S steel equal to ca. 600 MPa (Figure 14).

The comparison demonstrates a satisfactory agreement between the numerical predictions and the experimental data with a difference of 1.5%, confirming that the adopted modelling approach is capable of capturing the essential mechanical behaviour of the material.

### 3.3. Microstructural Cutting Simulation

The micro-scale cutting process of C75S steel was numerically investigated in order to analyse the mechanisms of material separation at the level of individual microstructural constituents. The simulations were performed using the finite element method implemented in LS-DYNA [22], with a particular emphasis on the interactions between the cutting tool and the heterogeneous microstructure composed of ferritic matrix, globular cementite, and interfacial zones.

#### 3.3.1. Evolution of Strain and Stress During Cutting Process

The objective of the analysis was to evaluate the evolution of equivalent Huber–Mises stress and strain during the cutting process and to identify the dominant mechanisms governing deformation, fracture, and phase interaction under localised loading conditions.

**Figure 15:**
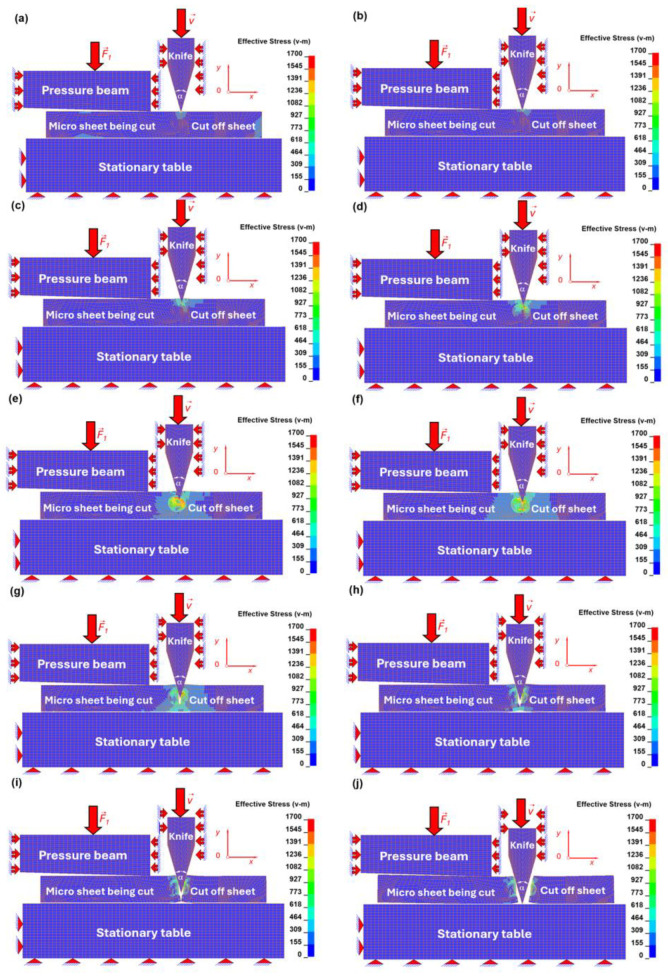
Effective stress (equivalent Huber–Mises stress) distribution stages in the micro sheet during the progressive cutting process for the chosen pseudo time instances [ms]: (**a**) t = 0.3, (**b**) t = 0.6, (**c**) t = 1.5, (**d**) t = 2, (**e**) t = 3, (**f**) t = 3.7, (**g**) t = 4.8, (**h**) t = 5.7, (**i**) t = 6.1, (**j**) t = 10.

**Figure 16:**
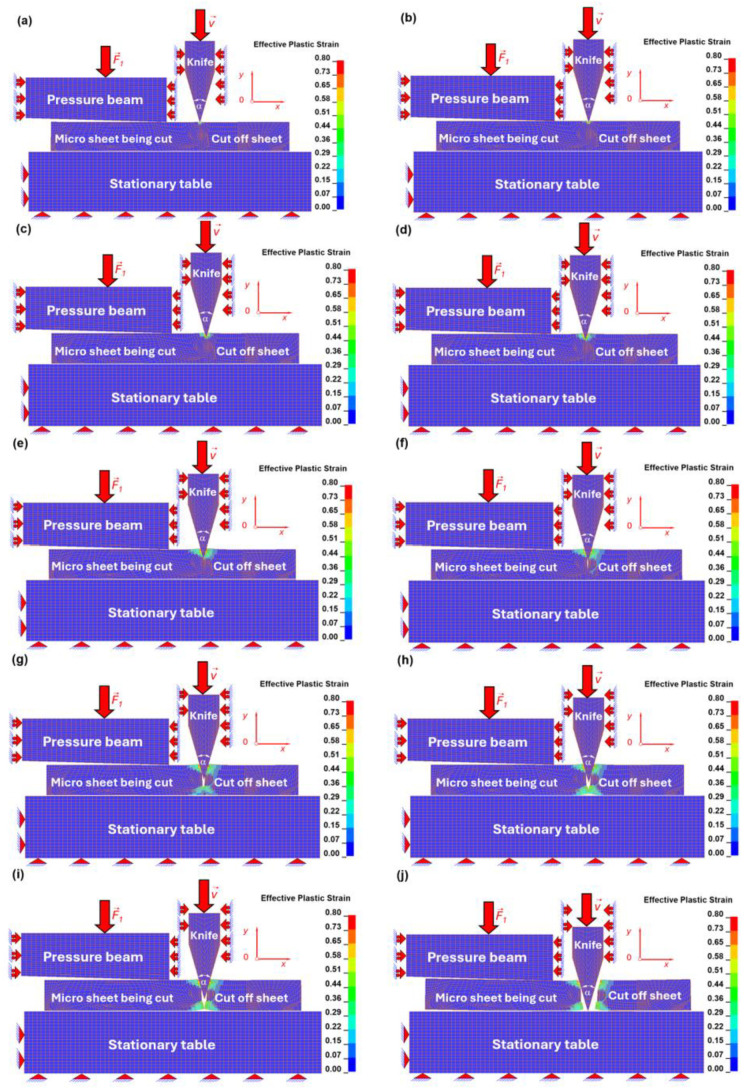
Effective plastic strain (equivalent Huber–Mises strain) distribution stages in the micro sheet during the progressive cutting process for the chosen pseudo time instances [ms]: (**a**) t = 0.3, (**b**) t = 0.6, (**c**) t = 1.5, (**d**) t = 2, (**e**) t = 3, (**f**) t = 3.7, (**g**) t = 4.8, (**h**) t = 5.7, (**i**) t = 6.1, (**j**) t = 10.

At the initial stage (Figure 15a and Figure 16a), the cutting tool comes into contact with the surface of the deformable sheet. The stress field is concentrated beneath the tool tip, while the strain remains relatively low, indicating the onset of elastic–plastic deformation.

In the subsequent stage (Figure 15b and Figure 16b), plastic deformation intensifies in the ferritic matrix directly under the tool. The equivalent strain exceeds the critical value assumed for the ferritic phase (ε ≈ 0.3), leading to node separation along the predefined cutting line (Figure 16b). This marks the initiation of material failure and allows for further penetration of the cutting tool. Correspondingly, a significant increase in equivalent stress is observed in the vicinity of the tool–material contact zone (Figure 15b).

As the tool advances (Figure 15c and Figure 16c), the deformation zone propagates towards the interfacial region. Due to the higher strength of the interface compared to the ferritic matrix, stress concentrations increase at the phase boundary (Figure 15c), while strain localisation becomes more confined (Figure 16c).

Further penetration of the tool (Figure 15d and Figure 16d) leads to interaction with the globular cementite particle. Owing to its brittle nature and lower failure strain (ε ≈ 0.01), the cementite grain begins to crack. This is reflected by a sharp increase in equivalent stress within the particle (Figure 15d), accompanied by localised strain accumulation at the crack initiation sites (Figure 16d).

In Figure 15e and Figure 16e, crack propagation within the cementite grain becomes more pronounced. The stress field redistributes as the particle fractures (Figure 15e), while high strain gradients develop along the crack path (Figure 16e). The brittle failure of cementite is characterised by relatively low strain but high stress levels.

Subsequently (Figure 15f and Figure 16f), the fracture of the cementite particle is completed, and the cutting process progresses into the surrounding interfacial zone. Elevated stress concentrations are again observed at the interface, indicating its role as a transitional region governing load transfer between phases (Figure 15f).

In Figure 15g and Figure 16g, the tool penetrates beyond the interface and re-enters the ferritic matrix. Plastic deformation becomes dominant once more, with increasing equivalent strain distributed over a larger tool penetration beneath the cutting tool tip (Figure 16g).

A significant change in the deformation mechanism is observed in Figure 15h and Figure 16h, where the material response transitions from shear-dominated cutting to tensile stretching. This is evidenced by the development of a necking region beneath the sheet, accompanied by a redistribution of stress (Figure 15h) and a marked increase in equivalent strain (Figure 16h).

In the final stages (Figure 15i,j as well as Figure 16i,j), complete separation of the material occurs. The equivalent strain reaches its maximum values in the necked region (Figure 16i,j), while the stress decreases locally due to material failure and loss of load-carrying capacity (Figure 15i,j). The cutting tool reaches the surface of the stationary table, finalising the cutting process, after which it returns to its initial position.

The obtained results demonstrate that the micro-scale cutting process in C75S steel is governed by a complex interaction between ductile deformation in the ferritic matrix and brittle fracture of cementite particles. The distributions of effective plastic strain (equivalent Huber–Mises strain) and effective stress (equivalent Huber–Mises stress) clearly indicate that:

- ferrite undergoes large plastic deformation prior to failure,
- the interface acts as a zone of stress concentration,
- cementite particles fail in a brittle manner under high stress and low strain,
- and the overall cutting mechanism evolves from shear-dominated to tensile-dominated fracture.

These findings provide an important insight into the micro-mechanisms of material separation and are directly relevant for understanding cutting processes at the micro-scale.

#### 3.3.2. Discussion of Microstructure Cutting Simulation

The results of the micro-scale cutting simulation of C75S steel reveal a strongly heterogeneous deformation and fracture behaviour governed by the interactions between the ferritic matrix, globular cementite particles, and the interfacial transition zones. The distributions of Huber–Mises stress and strain indicate that plastic deformation is predominantly localised in the ferritic matrix, while cementite particles sustain high stress levels and fail in a brittle manner. The ferrite–cementite interface plays a crucial role as a region of stress concentration and governs crack initiation and propagation. The results demonstrate that the presence, size, and distribution of cementite particles significantly influence the cutting behaviour and the evolution of deformation mechanisms at the micro-scale.

The cutting process is initially governed by a shear-dominated mechanism, which progressively evolves into a combined shear–tensile deformation mode and ultimately transitions to tensile-dominated stretching. This transition results in pronounced strain localisation and necking along the cutting line, culminating in final material separation through fracture. The detailed values of equivalent Huber–Mises stress and strain in the consecutive micro-cutting process are presented in Table 12.

The distribution of equivalent Huber–Mises stress and strain was analysed in the individual constituents of the microstructural model, namely the ferritic matrix, the interface, and the globular cementite particles, during the micro-cutting process.

In all three constituents, the maximum equivalent Huber–Mises stresses increase progressively with the advancement of the cutting process. The highest stress levels are observed in the cementite particles, reaching a maximum value of 1582 MPa. Lower values are recorded at the interface (680 MPa), while the lowest stresses occur in the ferritic matrix (430 MPa).

The corresponding equivalent Huber–Mises strains show an opposite trend, with the highest strain levels observed in the ferritic matrix (0.801), followed by the interface (0.332), and the lowest values in the cementite particles (0.0736), reflecting the differences in mechanical properties of the individual phases.

At the final stage of the cutting process, the remaining stress and strain fields can be interpreted as residual stresses and strains. In this state, the equivalent Huber–Mises stresses decrease but remain highest in cementite (1182 MPa), followed by the interface (539 MPa) and the ferritic matrix (346 MPa).

The corresponding residual equivalent strains are equal to 0.2272 in cementite, 0.463 at the interface, and 0.808 in the ferritic matrix. These results indicate that, while cementite carries the highest stresses, the ferritic matrix accommodates the largest plastic deformation, with the interface exhibiting intermediate behaviour.

## 4. Conclusions

The combined experimental and numerical investigations provide a comprehensive understanding of the influence of microstructure on the mechanical behaviour of C75S steel. The microstructural observations performed using scanning electron microscope and energy-dispersive X-ray spectroscopy confirmed the heterogeneous nature of the material, consisting of a ductile ferritic matrix and hard globular cementite particles.

The numerical simulations based on the finite element method within the LS-DYNA environment demonstrated that the mechanical response of the material is strongly governed by the interaction between these microstructural constituents. In the micro-scale tensile test, deformation was primarily accommodated by the ferritic matrix, while stress concentrations developed at the ferrite–cementite interfaces and within larger cementite particles.

The simulations of the micro-tensile test indicate two distinct damage mechanisms of crack initiation:

- interface-controlled cracking, occurring mainly for smaller cementite particles (from ca. 0.5 to 1.2 μm), where stress and strain concentrations develop along the ferrite–cementite boundaries;
- particle-controlled cracking, observed for larger cementite grains (from ca. 1.2 to 2.0 μm), where stress concentrations develop inside the cementite particle, leading to fracture within the brittle phase.

The comparison between the experimental and numerical true stress–true strain responses demonstrates quite a good agreement in terms of yield strength. The experimentally determined yield strength of 600 MPa is in very close correspondence with the numerically predicted value of 609 MPa. This negligible difference (1.5%) confirms the accuracy and reliability of the adopted numerical model in capturing the onset of plastic deformation. Consequently, the results validate the modelling approach and support its applicability for analysing the mechanical behaviour of the material at the micro-scale.

In the case of the micro-scale cutting process, the material response was characterised by a combination of ductile shearing in the ferritic matrix and brittle fracture of cementite particles. The interface played a crucial role as a zone of stress concentration and transition of deformation mechanisms. The cutting process evolved from shear-dominated deformation to tensile stretching, leading to final material separation accompanied by localised necking. The obtained results clearly demonstrate that the mechanical response during the cutting process is governed by the strong contrast in properties between the individual microstructural constituents. Cementite particles act as primary load-bearing elements, accumulating the highest stress levels (1582 MPa), while the ferritic matrix accommodates the majority of plastic strain (0.801). The interface plays a transitional role, facilitating stress transfer (680 MPa), and strain (0.332) compatibility between the phases. This interplay between stress concentration in hard particles and strain localisation in the soft matrix is a key factor controlling the overall deformation behaviour and potential damage initiation in the material.

Overall, the results indicate that the size, distribution, and mechanical contrast of cementite particles and ferritic matrix, as well as the properties of the interfacial zone, have a decisive impact on both tensile and cutting behaviour. The presented approach demonstrates that microstructural modelling is essential for accurately capturing deformation and fracture mechanisms, and it provides a reliable basis for further studies on material separation processes at the micro-scale.

## Figures and Tables

**Table 1:** Chemical composition of C75S high-carbon steel in weight percent.

Grade	C	Mn	P	S	Si	Cr	Mo	Ni
C75S	0.70–0.80	0.60–0.90	≤0.025	≤0.025	0.15–0.35	≤0.40	≤0.10	≤0.40

**Table 2:** Mechanical material properties for individual phases which have been assumed similarly like in the literature [27,28,29,42].

No	Individual Phase Properties	Young’s Modulus E [GPa]	Tangent Modulus E_T_ [GPa]	Poisson’s Ratio ν [-]	Yield Strength R_e_ [MPa]	Hardening Modulus H [MPa]	Strain Hardening Exponent n [-]
1.	Ferritic matrix	210	-	0.29	200	76	0.23
2.	Interface	215	-	0.28	500	64	0.10
3.	Globular cementite	200	5	0.27	1200	-	-

**Table 3:** Dimensions of the modelled sample.

Length [μm]	L_0_ = 16	L_1_ = 20
Height [μm]	H_0_ = 6	H_1_ = 8

**Table 4:** Finite element discretisation details of the analysed micro-tensile test model.

Component	Type	Number of Elements	Number of Nodes	Element Type
Deformable sheet (total)	Deformable	4817	4901	2D quadrilateral and triangular
Ferritic matrix	Deformable	3421	3661	2D quadrilateral and triangular
Globular cementite grains	Deformable	1046	1240	2D quadrilateral and triangular
Interface zone	Deformable	350	704	2D quadrilateral
Rigid grips (total)	Rigid	952	1050	2D rigid quadrilateral
Fixed grip	Rigid	448	495	2D rigid quadrilateral
Kinematic grip	Rigid	504	555	2D rigid quadrilateral
Total	Deformable and rigid	5769	5881	2D quadrilateral and triangular

**Table 5:** Selected geometrical features of globular cementite grains of C75S steel.

Grain’s Number According to Figure 2 and Figure 3	Area [μm^2^]	Centroid Along *x*-Axis [μm]	Centroid Along *y*-Axis [μm]
1	0.826	9.851	5.166
2	2.766	10.255	1.662
3	1.053	1.851	0.896
4	0.585	4.523	1.091
5	0.209	1.156	3.934
6	0.154	14.663	2.795
7	1.413	13.973	4.369
8	2.497	6.380	1.507
9	5.320	4.815	4.426
10	3.135	14.754	0.670
Total	17.957	8.416	2.633

**Table 6:** Selected geometrical features of structural constituents of C75S steel.

No	Structural Constituent According to Figure 2 and Figure 3	Area [μm^2^]	Centroid Along *x*-Axis [μm]	Centroid Along *y*-Axis [μm]	Percentage Contribution of Individual Constituents [%]
1	Globular cementite	17.957	8.416	2.633	18.71
2	Ferritic matrix	73.306	7.896	3.111	76.36
3	Interface	4.738	8.037	2.676	4.93
4	Total	96.001	8	3	100

**Table 7:** Finite element discretisation details of the analysed micro-cutting model.

Component	Type	Number of Elements	Number of Nodes	Element Type
Deformable sheet (total)	Deformable	1337	1441	2D quadrilateral and triangular
Ferritic matrix	Deformable	957	1085	2D quadrilateral and triangular
Globular cementite grains	Deformable	292	356	2D quadrilateral and triangular
Interface zone	Deformable	88	188	2D quadrilateral
Rigid components (total)	Rigid	2300	2478	2D rigid quadrilateral
Stationary table	Rigid	1400	1491	2D rigid quadrilateral
Pressure beam	Rigid	600	651	2D rigid quadrilateral
Cutting tool	Rigid	300	336	2D rigid quadrilateral
Total	Deformable and rigid	3637	3919	2D quadrilateral and triangular

**Table 8:** Quantitative results for a point located inside a globular cementite particle.

Element Line	Net Counts	Net Counts Error	Weight %	Weight % Error	Atom %	Atom % Error	Formula	Compnd %	# Cations
Cr K	1632	+/- 81	1.32	+/- 0.07	1.41	+/- 0.07	Cr	1.32	---
Mn K	2848	+/- 202	3.61	+/- 0.26	3.66	+/- 0.26	Mn	3.61	---
Fe K	70,973	+/- 463	95.08	+/- 0.62	94.93	+/- 0.62	Fe	95.08	---
Total			100.00		100.00			100.00	0.000

**Table 9:** Quantitative results for a small rectangular region representing the ferritic matrix.

Element Line	Net Counts	Net Counts Error	Weight %	Weight % Error	Atom %	Atom % Error	Formula	Compnd %	# Cations
Fe K	78,789	+/- 471	100.00	+/- 0.60	100.00	+/- 0.60	Fe	100.00	---
Total			100.00		100.00			100.00	0.000

**Table 10:** Quantitative results for a small rectangular region corresponding to globular cementite.

Element Line	Net Counts	Net Counts Error	Weight %	Weight % Error	Atom %	Atom % Error	Formula	Compnd %	# Cations
Mn K	645	+/- 83	0.76	+/- 0.10	0.77	+/- 0.10	Mn	0.76	---
Fe K	79,918	+/- 479	99.24	+/- 0.59	99.23	+/- 0.59	Fe	99.24	---
W L	0	+/- 41	0.00	---	0.00	+/- 0.00	W	0.00	---
Total			100.00		100.00			100.00	0.000

**Table 11:** Maximum values of stress and strain for individual phases of C75S steel in progressive pseudo time intervals according to Figure 11 and Figure 12.

Pseudo Time t (According to Figure 11 and Figure 12) [s]	Ferritic Matrix		Interface		Globular Cementite	
	Max Stress [MPa]	Max Strain [-]	Max Stress [MPa]	Max Strain [-]	Max Stress [MPa]	Max Strain [-]
0.2	390	0.224	592	0.081	11	0.001
0.4	444	0.395	653	0.193	1378	0.019
0.6	462	0.536	683	0.301	1534	0.044
0.8	470	0.708	696	0.447	1624	0.068
1.0	478	0.866	703	0.661	1655	0.084

**Table 12:** Maximum values of stress and strain for individual phases of C75S steel in micro-cutting process according to Figure 15 and Figure 16.

Stages of Progress of Cutting Process According to Figure 15 and Figure 16	Ferritic Matrix		Interface		Globular Cementite	
	Max Stress [MPa]	Max Strain [-]	Max Stress [MPa]	Max Strain [-]	Max Stress [MPa]	Max Strain [-]
(a)	384	0.287	303	0	218	0
(b)	423	0.482	118	0	98	0
(c)	427	0.482	578	0.094	499	0.0004
(d)	401	0.695	680	0.332	1582	0.0736
(e)	430	0.801	525	0.462	1527	0.2272
(f)	400	0.807	567	0.463	1301	0.2272
(g)	424	0.808	672	0.463	1566	0.2274
(h)	405	0.808	559	0.463	1262	0.2272
(i)	347	0.808	537	0.463	1162	0.2272
(j)	346	0.808	539	0.463	1182	0.2272

## Data Availability

The original contributions presented in this study are included in the article. Further inquiries can be directed to the corresponding author.
